# Effects of statins and aspirin on HCC risk in alcohol-related cirrhosis: nationwide emulated trials

**DOI:** 10.1097/HC9.0000000000000013

**Published:** 2023-01-03

**Authors:** Frederik Kraglund, Diana H. Christensen, Andreas H. Eiset, Gerda E. Villadsen, Joe West, Peter Jepsen

**Affiliations:** 1 Department of Hepatology and Gastroenterology, Aarhus University Hospital, Aarhus, Denmark; 2 Department of Clinical Medicine, Aarhus University, Aarhus, Denmark; 3 Department of Clinical Epidemiology, Aarhus University Hospital, Aarhus, Denmark; 4 Department of Affective Disorders, Aarhus University Hospital, Aarhus, Denmark; 5 Lifespan and Population Health, School of Medicine, University of Nottingham, Nottingham, UK; 6 NIHR Biomedical Research Unit, Nottingham University Hospitals NHS Trust and University of Nottingham, Nottingham, UK

## Abstract

Observational studies have shown an association between statin or aspirin use and a decreased risk of HCC, but the effects of a well-defined treatment strategy remain unknown. We emulated trials of the effects of continuous statin or aspirin use on HCC risk in patients with cirrhosis due to alcohol-related liver disease (ALD cirrhosis).

**Approach and Results:**

We specified target trials for statins and, separately, aspirin and emulated them using Danish health care registries. All eligible patients with ALD cirrhosis diagnosed in 2000–2018 were included in either an exposed or an unexposed arm. Patients were followed until HCC or death without HCC. The 5-year risk of HCC was estimated using marginal structural models with inverse probability weighting. Using statins continuously for 5 years compared with not using statins resulted in a relative risk (RR) of HCC of 0.67 (95% CI: 0.45–0.91). The RR of death without HCC was 0.69 (95% CI: 0.65–0.77). For aspirin, the RR was 1.05 (95% CI: 0.60–1.42) for HCC and 1.02 (95% CI: 0.95–1.09) for death without HCC.

**Conclusions:**

In patients with ALD cirrhosis, 5 years of continuous statin use resulted in a 33% RR reduction of HCC (number needed to treat = 94) and a 31% RR reduction of death without HCC (number needed to treat = 7). Such strong causal effects are implausible and best explained by uncontrollable confounding, highlighting the need for randomized trials. Aspirin use likely does not affect the risk of HCC or death without HCC.

## INTRODUCTION

HCC in patients with cirrhosis due to alcohol-related liver disease (ALD cirrhosis) has a very poor prognosis.^1^ Therefore, in reducing HCC-attributable mortality, there is potential to be gained from the prevention of HCC, for example, with chemopreventive drugs. Statins and aspirin use have been associated with a lower risk of HCC in numerous observational studies.^2^,^3^ We know from these studies that (1) patients who *have* developed HCC have a low probability of having ever used statins or aspirin,^4^,^5^ (2) patients who *initiate* treatment with statins or aspirin have a lower risk of HCC whether they choose to stay on the treatment or not,^6^,^7^ and (3) patients who are *currently* using statins or aspirin have a lower instantaneous risk of HCC.^8^,^9^ However, none of these associations answer the most relevant clinical question, which is this: How much can I reduce my patient’s risk of getting HCC by treatment with statins (or aspirin) for, say, five years? This question could be answered with a per-protocol analysis of a randomized controlled trial (RCT), but so far no RCT on the effect of statins or aspirin on HCC risk has been published. Furthermore, a per-protocol analysis of an RCT is effectively an observational study confounded by differential adherence to protocol.^10^ Emulating a target trial is a causal inference school of thought, wherein the aim is to imitate an RCT as closely as possible using observational data. The method helps to minimize common biases of observational studies, that is, studies that emulate a target trial provide results that are closer to those from randomized trials and are therefore more reliable.^11^

We emulated target trials of the chemopreventive effects of initiating and staying on statins and aspirin in patients with ALD cirrhosis.

## PATIENTS AND METHODS

### Target trials

We specified a target trial protocol for statins (and, separately, aspirin) and then designed its observational analog to emulate the target trial as closely as possible (Table 1). This method is described in detail by Dickerman et al.^12^ In the target trial, we would have included all Danish patients with ALD cirrhosis who met all these criteria: (1) diagnosed with ALD cirrhosis >6 months ago, (2) no use of statins (aspirin) within the last 12 months, (3) no history of HCC, (4) 30–89 years old, (5) no absolute contraindications to initiate treatment with statins (aspirin), and (6) diagnosed with ALD cirrhosis after 31 December 1999. For statins, the concurrent use of Glecaprevir/Pibrentasvir would have been an absolute contraindication, and for aspirin, coagulation disorders and gastrointestinal bleeding within the preceding 6 months would have been absolute contraindications.

**TABLE 1:** Target trial protocol and observational analog

	Target trial	Emulation
Eligibility criteria	(1) ALD cirrhosis diagnosed between 6 months and 10 years and 6 months ago, (2) ALD cirrhosis diagnosed no earlier than 1 January 2000, (3) no history of HCC, (4) no use of the drug of interest within the last year, (5) age between 30 and 89 years, (6) no absolute contraindication for the trial drug	Same as for target trial except for criterion (4) no use of the drug of interest within the year leading up to the 6 months before inclusion
Treatment allocation	Patients will be randomized to either (A) initiate and stay on the treatment of interest for 5 years, or (B) not initiate the treatment for 5 years	Eligible patients who initiate treatment with the trial drug within the 6 months leading up to inclusion will be included in the exposed arm (A), and patients who do not initiate treatment within the same 6-month period will be included in the unexposed arm (B)
Assignment procedures	Patients will be aware of the study and their assigned treatment	Patients will be aware of the their assigned treatment
Follow-up period	Starts at inclusion and ends at diagnosis of HCC, death, administrative censoring, or 5 years after baseline, whichever occurs first	Same as for target trial
Outcomes	HCC and death without HCC	Same as for target trial
Causal contrasts of interest	Per-protocol effect adjusted for time-varying factors associated with adherence to protocol	Per-protocol effect adjusted for baseline factors associated with drug initiation and time-varying factors associated with adherence to protocol
Analysis plan	5-year risk of HCC, 5-year relative risk of HCC, number needed to treat with the drug for 5 years to prevent 1 HCC. In the analyses of HCC, death without HCC will be regarded as a competing event and vice versa	Same as for target trial

Abbreviations: ALD cirrhosis, cirrhosis due to alcohol-related liver disease.

The target trial further specifies that all included patients would have been randomized to 1 of 2 five-year treatment strategies: (A) initiate and stay on statins (aspirin), or (B) do not use statins (aspirin). There would have been no blinding of the assigned treatment arm. The patients would have been followed until death or diagnosis of HCC. Event-free patients would have been censored administratively 5 years after randomization or on 31 December 2018, whichever occurred first. The following paragraphs describe how we used our registry data to emulate that target trial of statin (aspirin) use.

### Setting

This registry-based study was conducted in the Danish population of 5,806,081 people (1 January 2019). In Denmark, all citizens have access to free, tax-supported health care, and the cost of prescribed statins and aspirin is shared by the patient (with a per annum cap) and a tax-financed reimbursement system. The National Patient Registry contains data from all inpatient hospital contacts since 1977 and all outpatient and emergency room contacts since 1995.^13^ The Danish Cancer Registry records reported cancer cases since 1943, and since 1987 it has been obligatory to report all cancer cases in Denmark.^14^ The Danish Registry of Reimbursed Prescriptions contains data on all reimbursed prescriptions since 1997.^15^ The Danish Registry of Causes of Death records and immediate, underlying, and supplementary causes of death.^16^ According to the Danish Data Protection Act, studies based on data from Danish healthcare registries do not require approval from an ethics committee or written consent. HCC surveillance is not offered to Danish outpatients with ALD cirrhosis, because the annual HCC risk (0.7%) is lower than the threshold for surveillance efficacy (1.5%).^17^,^18^

### Study population

All patients with a first-time diagnosis of ALD cirrhosis in the study period from 1 January 2000 to 31 December 2018 were identified in the National Patient Registry using the 10th revision of the International Classification of Diseases (ICD-10) codes K70.3x. All incident HCC diagnoses during the study period were identified in the Cancer Registry and in the National Patient Registry using the ICD-10-codes C22.0x. All reimbursed statin and aspirin prescriptions were identified in the Registry of Reimbursed Prescriptions using the Anatomical Therapeutic Chemical (ATC) codes C10AAx, C10BAx, C10BXx (statins), and B01AC06 (aspirin). Causes of death were categorized by the underlying cause of death as either “liver-related” (ICD-10: F10x, I85x, I86x, K7x, R18x, X45x), “cardiovascular” (ICD-10: I20x-I25x, I27.2x, I51.3x, I63x [excl. I63.2x and I63.5x], I67.2x, I70x, I74x, H34.1A, K55.0C, K55.0H, K55.1A, N28.0A, N28.0D, T81.7B, T82.3D, T82.8A, Z86.7B), or “other.”

### Exposure to the trial drug

The total number of tablets handed out by the pharmacy or hospital was calculated as the number of tablets in a given package multiplied by the number of purchased packages. The number of days on the prescribed drug was approximated as the total number of tablets divided by the number of tablets the patient was to take per day according to the dosage instruction provided by the prescribing physician. In cases of missing dosage instructions, we assumed a daily dosage of one tablet. Patients were considered exposed to statins (aspirin) until 7 days after they had run out of tablets. Patients were considered adherent to the trial protocol if they filled a new prescription for statins (aspirin) within double the number of days of tablets specified by the last prescription. This definition was chosen to allow for patients forgetting to renew their prescriptions, forgetting to take the drug every day, or taking only half a tablet per day. For instance, if the last prescription was for 30 tablets taken once daily, the patient was considered exposed for 37 days and adherent to the protocol for 60 days. In the control arm, on the other hand, filling a prescription for statins (aspirin) was an immediate protocol deviation.

### Confounders

We chose confounders based on subject matter expertise, existing literature, and directed acyclic graphs of the effects of statins (aspirin) on the risk of HCC and death without HCC (Supplementary Figures S1 and S2, http://links.lww.com/HC9/A38).^19^ The included confounders are listed in Supplementary Tables S1 and S2 (http://links.lww.com/HC9/A38) along with the codes used to identify them in the registries. Briefly, we included sex, age, calendar year, time since first cirrhosis diagnosis, cumulative prior use of the trialed drug, hospital contacts and liver imaging examinations, cirrhosis decompensation, indications and relative contraindications for use of the trialed drug, common side effects of the trialed drug, potentially confounding drugs (including aspirin in the statin study and vice versa), and relapse of hazardous alcohol use. The included confounders were used to adjust for both statin (aspirin) initiation at baseline and statin (aspirin) adherence over time.

### Emulation design

We emulated the target trials 20 times for each trial drug to improve the precision in the estimates of association between statin (aspirin) use and the risk of HCC and death without HCC.^12^ This was achieved by creating 20 sequential trials starting 6 months apart, each running for 5 years. The starting point of each trial was defined by patient-time since the first ALD cirrhosis diagnosis to achieve an equal distribution of times since diagnosis. The first trial started 6 months after a patient’s first ALD cirrhosis diagnosis, and the last trial started 10 years later. For each trial, patients who met the target trial eligibility criteria at that time were included. They were assigned to treatment strategy A (exposed) or B (unexposed) based on their statin (aspirin) exposure or nonexposure during the 6-month period leading up to each trial. Thus, patients who began taking statins (aspirin) during the 6-month period leading up to the trial and were still exposed at the trial start date were included in the exposed arm, and the remaining eligible patients (i.e., patients who had not used statins [aspirin] during the previous year) were included in the unexposed arm (Supplementary Figure S3, http://links.lww.com/HC9/A38). Patients could be included in >1 trial if they began taking statins (aspirin) on multiple occasions separated by >1 year. The follow-up period, outcomes, and analysis plan were identical to those specified in the target trials (Table 1).

### Statistical analysis

Marginal structural models were used to estimate the causal effect of 5 years of continuous use of statins (aspirin) compared with 5 years of nonuse on the risk of HCC and on the risk of death without HCC.

In place of the target trial randomization, inverse probability of treatment weighting (IPTW) was used to create a pseudopopulation with balanced baseline confounders. Variables with standardized mean differences (SMDs) below 0.1 were considered well-balanced.^20^ To construct the marginal structural model, the continuous follow-up time was discretized at half-year intervals. To minimize protopathic bias, for example, bias by symptoms of undiagnosed HCC influencing the decision to stop treatment, we applied a lag-time in censoring by protocol deviation as follows^21^: patients in the exposed arm who stopped treatment by our definition of protocol adherence (60 days in the example used above) and patients in the unexposed arm who started treatment were censored after the next time interval (Supplementary Figure S3, http://links.lww.com/HC9/A38). To adjust for bias resulting from informative censoring, we used time-varying inverse probability of censoring weighting (IPCW) based on measured confounders.^22^ For both IPTW and IPCW, stabilized weights were used,^23^ and all probabilities used to derive the weights were computed using logistic regression.

The 5-year cumulative risk of HCC and of death without HCC was computed using pooled logistic regression weighted by baseline IPTW and time-varying IPCW, and also adjusted for baseline confounders (doubly robust estimation^24^). The 5-year relative risk (RR) was derived as the cumulative risk in the exposed arm divided by the cumulative risk in the unexposed arm. We calculated the number needed to treat from the cumulative risk differences, making the assumption that the observed differences were causal. Death without HCC was regarded as a competing event in the analyses of HCC risk and vice versa. Nonparametric cluster bootstrapping with 500 replications was used to compute percentile-based 95% CIs. For a detailed description of the statistical methods used, see the Supplementary Methods (http://links.lww.com/HC9/A38).

### Sensitivity analyses

To assess whether the study design or confounder model had residual bias, we repeated the analyses with the following “negative control” outcomes:^25^ non-HCC cancer (ICD-10: Cx [excl. C22.0x]), lung cancer (ICD-10: C34x), and fractures likely to be caused by low-energy trauma (ICD-10: S32.1-S32.4, S72x [excl. S72.9], S52.0x, S624, S82.1-S82.7, S92.0x, S92.3x, S42.2x-S42.4x).^26^ In addition, we assessed the risk of acute myocardial infarction or ischemic stroke (ICD-10: I21x, I63x) in the trial arms. We also repeated the primary analyses excluding the first year of follow-up to account for the delay between HCC development and HCC diagnosis. Last, we investigated the effects of statins (aspirin) on liver-related deaths, cardiovascular deaths, and other deaths in a competing risk setting.

## RESULTS

### Statins

We included 1438 (1351 distinct) patients with ALD cirrhosis in the exposed arms and 118,460 (14,653 distinct) patients with ALD cirrhosis in the unexposed arms of the 20 statin trials. Unweighted and weighted baseline characteristics are presented with SMDs in Table 2. After weighting, all confounders were well-balanced with SMDs below 0.1. During 369,003 person-years of follow-up, 3101 patients were diagnosed with HCC, 47,015 died without HCC, 7991 were censored by protocol deviation, and the remaining 61,791 were administratively censored after 5 years of follow-up or on 31 December 2018. The overall 5-year risk of HCC was 3.1%, and the 5-year risk of death without HCC was 46%.

**TABLE 2:** Baseline characteristics of patients in the statin trials; observed population, and weighted pseudopopulation with SMDs

	Observed population			Reweighted pseudopopulation		
	Statin arm (N = 1438), n (%)	Control arm (N = 118,460), n (%)	SMD	Statin arm (N = 1376), n (%)	Control arm (N = 118,522), n (%)	SMD
Male sex	965 (67.1)	76,716 (64.8)	0.050	884 (64.3)	76,788 (64.8)	−0.011
Age group (years)						
30–49	122 (8.5)	18,324 (15.5)	−0.216	186 (13.5)	18,233 (15.4)	−0.053
50–59	434 (30.2)	41,666 (35.2)	−0.107	467 (33.9)	41,615 (35.1)	−0.025
60–69	602 (41.9)	41,347 (34.9)	0.143	501 (36.4)	41,468 (35.0)	0.029
≥70	280 (19.5)	17,123 (14.5)	0.134	223 (16.2)	17,204 (14.5)	0.046
Period of eligibility, median (IQR)						
2000–2005	123 (8.6)	20,171 (17.0)	−0.256	188 (13.6)	20,060 (16.9)	−0.091
2006–2011	593 (41.2)	44,098 (37.2)	0.082	516 (37.5)	44,176 (37.3)	0.005
2012–2018	722 (50.2)	54,191 (45.8)	0.089	672 (48.8)	54,285 (45.8)	0.061
Cumulative days of statin exposure before inclusion						
0–34	1078 (75.0)	108,650 (91.7)	−0.461	1241 (90.2)	108,464 (91.5)	−0.047
35–141	72 (5.0)	2395 (2.0)	0.163	30 (2.2)	2439 (2.1)	0.010
142–425	83 (5.8)	2502 (2.1)	0.189	39 (2.8)	2557 (2.2)	0.043
426–1113	87 (6.1)	2488 (2.1)	0.201	32 (2.4)	2546 (2.2)	0.014
≥1113	118 (8.2)	2425 (2.1)	0.282	34 (2.5)	2515 (2.1)	0.022
Hospital contacts, median (IQR)	0 (0–2)	0 (0–2)	−0.053	0 (0–2)	0 (0–2)	−0.005
Liver imaging examination	64 (4.5)	4937 (4.2)	0.014	63 (4.6)	4944 (4.2)	0.019
Acute ALD cirrhosis diagnosis	845 (58.8)	69,626 (58.8)	−0.000	804 (58.4)	69,663 (58.8)	−0.007
Decompensated cirrhosis	623 (43.3)	58,028 (49.0)	−0.114	652 (47.4)	57,977 (48.9)	−0.031
Kidney insufficiency	56 (3.9)	3028 (2.6)	0.076	43 (3.1)	3049 (2.6)	0.032
Hyperlipidemia	132 (9.2)	3189 (2.7)	0.277	52 (3.8)	3284 (2.8)	0.056
Obesity	209 (14.5)	11,522 (9.7)	0.148	158 (11.5)	11,598 (9.8)	0.056
Hypertension	907 (63.1)	59,370 (50.1)	0.264	726 (52.8)	59,588 (50.3)	0.050
Diabetes	399 (27.7)	14,401 (12.2)	0.398	200 (14.6)	14,634 (12.3)	0.065
Cardiovascular disease	303 (21.1)	10,297 (8.7)	0.353	136 (9.8)	10,481 (8.8)	0.035
Dyspepsia	1046 (72.7)	82,144 (69.3)	0.075	978 (71.0)	82,236 (69.4)	0.036
Myalgia	98 (6.8)	6410 (5.4)	0.059	82 (6.0)	6434 (5.4)	0.024
Metformin	103 (7.2)	1531 (1.3)	0.295	24 (1.7)	1618 (1.4)	0.029
Aspirin	174 (12.1)	2858 (2.4)	0.380	40 (2.9)	3000 (2.5)	0.022
Hazardous alcohol use relapse	30 (2.1)	710 (0.6)	0.129	8 (0.6)	731 (0.6)	−0.000

Abbreviations: ALD cirrhosis, cirrhosis due to alcohol-related liver disease; IQR, interquartile range; SMD, standardized mean difference.

**FIGURE 1:**
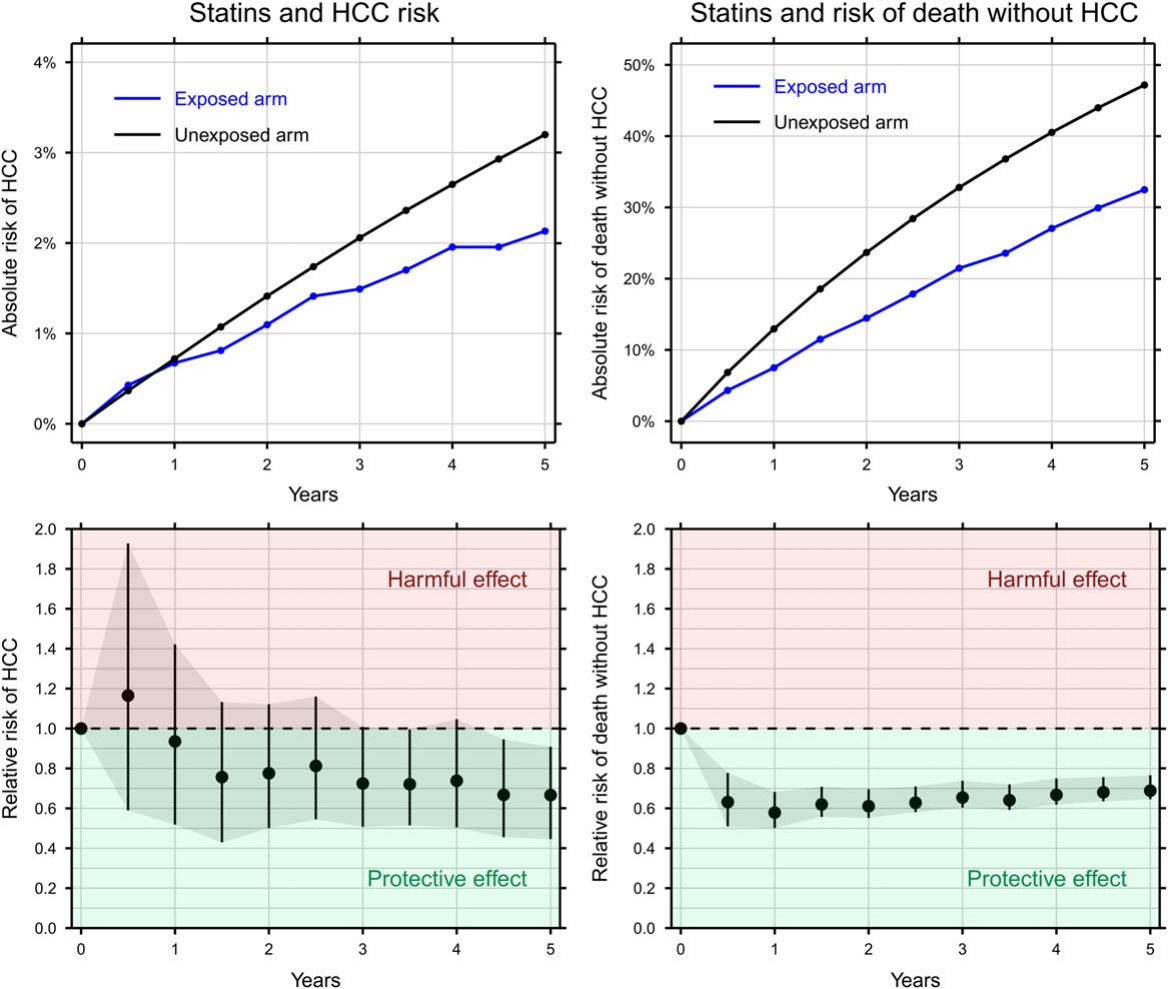
The effect of statins on HCC (left) and death without HCC (right) shown as the cumulative incidence (top) and relative risk with bootstrapped 95% CIs.

The 5-year risk of HCC was 2.1% in the statin arm and 3.2% in the control arm corresponding to a RR of 0.67 (95% CI: 0.45–0.91). By extension, the number of patients needed to treat with statins for 5 years to prevent 1 HCC was 94. The 5-year risk of death without HCC was 32% in the statin arm and 47% in the control arm corresponding to a RR of 0.69 (95% CI: 0.65–0.77). The effect was swift, and the RR of death without HCC was 0.63 (95% CI: 0.51–0.78) after just 6 months. The number of patients needed to treat with statins for 5 years to prevent 1 death without HCC was 7 (Figure 1).

### Aspirin

We included 1449 (1357 distinct) patients in the exposed arms and 113,643 (14,288 distinct) patients in the unexposed arms of the 20 aspirin trials. All confounders were well-balanced with SMDs below 0.1 (Table 3). During 358,454 person-years of follow-up, 2830 patients were diagnosed with HCC, 41,978 died without HCC, 7130 were censored by protocol deviation, and the remaining 63,154 were administratively censored after 5 years of follow-up or on 31 December 2018. The overall 5-year risk of HCC was 3.0%, and the 5-year risk of death without HCC was 44%.

**TABLE 3:** Baseline characteristics of patients in the aspirin trials; observed population, and weighted pseudopopulation with SMDs

	Observed population			Reweighted pseudopopulation		
	Aspirin arm (N = 1449), n (%)	Control arm (N = 113,643), n (%)	SMD	Aspirin arm (N = 1449), n (%)	Control arm (N = 113,643), n (%)	SMD
Male sex	993 (68.5)	72,773 (64.0)	0.095	933 (64.4)	72,838 (64.1)	0.007
Age group (years)						
30–49	95 (6.6)	17,262 (15.2)	−0.280	204 (14.1)	17,138 (15.1)	−0.027
50–59	410 (28.3)	39,867 (35.1)	−0.146	498 (34.4)	39,769 (35.0)	−0.013
60–69	640 (44.2)	39,769 (35.0)	0.188	510 (35.2)	39,900 (35.1)	0.002
≥70	304 (21.0)	16,745 (14.7)	0.164	236 (16.3)	16,836 (14.8)	0.042
Period of eligibility, median (IQR)						
2000–2005	263 (18.2)	17,935 (15.8)	0.063	179 (12.4)	17,966 (15.8)	−0.099
2006–2011	615 (42.4)	41,206 (36.3)	0.127	506 (35.0)	41,295 (36.3)	−0.029
2012–2018	571 (39.4)	54,502 (48.0)	−0.173	763 (52.7)	54,382 (47.9)	0.096
Cumulative days of aspirin exposure before inclusion						
0–106	1079 (74.5)	101,661 (89.5)	−0.398	1260 (86.9)	101,445 (89.3)	−0.072
107–362	172 (11.9)	5998 (5.3)	0.237	79 (5.4)	6092 (5.4)	0.003
363–1153	112 (7.7)	2984 (2.6)	0.232	49 (3.4)	3058 (2.7)	0.039
≥1154	86 (5.9)	3000 (2.6)	0.163	62 (4.3)	3049 (2.7)	0.088
Hospital contacts, median (IQR)	1 (0–3)	0 (0–2)	−0.097	0 (0–2)	0 (0–2)	−0.022
Liver imaging examination	61 (4.2)	4707 (4.1)	0.003	63 (4.3)	4708 (4.1)	0.009
Acute ALD cirrhosis diagnosis	859 (59.3)	66,399 (58.4)	0.017	847 (58.5)	66,411 (58.4)	0.000
Decompensated cirrhosis	560 (38.6)	55,236 (48.6)	−0.202	716 (49.4)	55,093 (48.5)	0.019
Kidney insufficiency	61 (4.2)	3077 (2.7)	0.082	43 (3.0)	3099 (2.7)	0.016
Hypertension	914 (63.1)	57,154 (50.3)	0.260	723 (49.9)	57,338 (50.5)	−0.011
Diabetes	320 (22.1)	15,489 (13.6)	0.222	228 (15.7)	15,612 (13.7)	0.056
Cardiovascular disease	330 (22.8)	9253 (8.1)	0.413	130 (9.0)	9463 (8.3)	0.023
Heart failure	132 (9.1)	3330 (2.9)	0.262	52 (3.6)	3419 (3.0)	0.034
Heart arrhythmia	200 (13.8)	7048 (6.2)	0.255	113 (7.8)	7160 (6.3)	0.060
Dyspepsia	1005 (69.4)	78,954 (69.5)	−0.003	1010 (69.7)	78,953 (69.5)	0.005
Anemia	206 (14.2)	14,549 (12.8)	0.041	185 (12.8)	14,570 (12.8)	−0.002
Metformin	67 (4.6)	2233 (2.0)	0.150	35 (2.4)	2269 (2.0)	0.030
Statins	154 (10.6)	3919 (3.4)	0.283	56 (3.9)	4022 (3.5)	0.018
Antithrombogenic drugs (except aspirin)	120 (8.3)	3041 (2.7)	0.248	50 (3.5)	3122 (2.7)	0.042
Hazardous alcohol use relapse	30 (2.1)	1780 (1.6)				

Abbreviations: ALD cirrhosis, cirrhosis due to alcohol-related liver disease; IQR, interquartile range; SMD, standardized mean difference.

**FIGURE 2:**
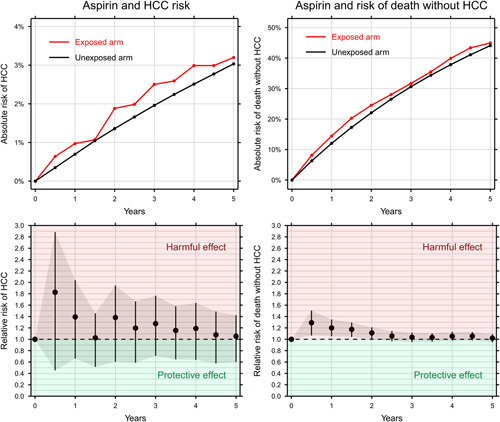
The effect of aspirin on HCC (left) and death without HCC (right) shown as the cumulative incidence (top) and relative risk with bootstrapped 95% CIs.

The 5-year risk of HCC was 3.2% in the aspirin arm and 3.0% in the control arm corresponding to a RR of 1.05 (95% CI: 0.60–1.42), meaning that 5 years of continuous aspirin use did not affect the risk of HCC. The 5-year risk of death without HCC was 45% in the aspirin arm and 44% in the control arm corresponding to a RR of 1.02 (95% CI: 0.95–1.09) (Figure 2).

### Sensitivity analyses

**FIGURE 3:**
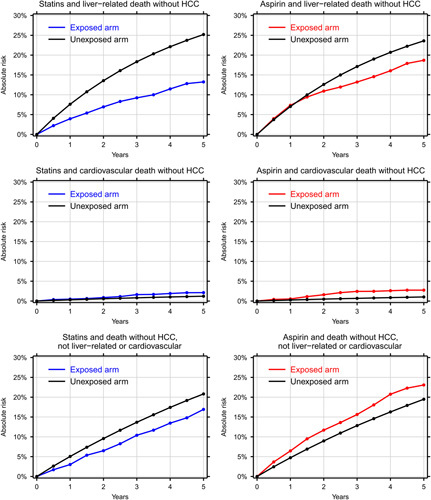
The effect of statins (left) and aspirin (right) on the cumulative incidence of liver-related death without HCC (top), cardiovascular death without HCC (middle), and death without HCC attributed to other causes (bottom).

Our sensitivity analyses showed that neither statins nor aspirin had a notable association with the risk of non-HCC cancer or lung cancer. For the first 2 years of continued statin use, statin users and nonusers had the same risk of fractures commonly caused by low-energy trauma, but after that time, statin users had a lower risk of those fractures. Aspirin users, by contrast, had a relatively high risk of fractures commonly caused by low-energy trauma and consequently a notably higher risk than statin users (5-year risk = 9% among statin users vs. 15% among aspirin users). Statin users and aspirin users had similarly increased risks of acute myocardial infarction or ischemic stroke compared with patients in the unexposed arms (Supplementary Figures S4 and S5, http://links.lww.com/HC9/A38). Excluding the first year of follow-up did not significantly change the effect size of statins (aspirin) on the risk of HCC or death without HCC (Supplementary Figure S6, http://links.lww.com/HC9/A38). Regarding causes of death, 5 years of statin use was associated with an increased risk of cardiovascular death (absolute risk: 2.1% vs. 1.2%), a marked decrease in liver-related death (13% vs. 25%), and a slight decrease in deaths attributed to other causes (17% vs. 21%). Five years of aspirin use was associated with an increased risk of cardiovascular death (2.7% vs. 1.0%), a slightly decreased risk of liver-related death (19% vs. 24%), and a slightly increased risk of death attributed to other causes (23% vs. 19%) (Figure 3).

## DISCUSSION

The number of deaths from HCC has increased dramatically in recent decades,^27^ and chemoprevention of HCC among people with cirrhosis is an attractive approach to curb this increase. Our emulated trials examined the effects of statins and aspirin on the risk of HCC in patients with ALD cirrhosis. We found no effect of aspirin, but a substantial effect of statins on not only HCC development (33% risk reduction after 5 years, number needed to treat = 94) but also on death without HCC (31% risk reduction, number needed to treat = 7). The burning question is whether these apparent effects of statins are too good to be true.

The reliance on data from registries is a potential limitation of our study, but incorrect codes cannot explain our findings. The codes for liver cirrhosis were validated in 1997 (N = 198) in Denmark with a high positive predictive value of 85.4% (95% CI: 79.8%–89.6%) and completeness of 93.2% (85.9%–96.8%),^28^ and the codes for liver diseases, in general, were validated again in 2011 (N = 100) with positive predictive values of 100% (95% CI: 92.9%–100%).^29^ The Danish Cancer Registry generally has a very high validity and completeness owing to rigorous validation and mandatory reporting of incident cancers to the registry.^30^ The codes for statin prescriptions have been validated by blood samples with a positive predictive value of 93% (95% CI: 86%–97%) and a negative predictive value of 93% (95% CI: 86%–97%),^31^ and the codes for low-dose aspirin have also been validated with a prevalence of misclassification of true aspirin use as nonuse of only around 1% in the hospital setting.^32^ Moreover, it is exceedingly unlikely that the validity of diagnosis codes depends on the patient’s propensity to use statins or aspirin. Also, neither statins nor low-dose aspirin are sold without a prescription in Denmark. Therefore, we should have near-complete, unbiased ascertainment of diagnosed ALD cirrhosis, HCC and the prescribed drugs of interest.

We believe that the apparent effects of statin use we have observed are likely too good to be true and that they are partly or fully explained by uncontrolled confounding. First, observational studies cannot control for unmeasured and unmeasurable confounders.^33^ This limitation was evident from the apparent residual “confounding by indication” in our sensitivity analyses, in which both statin and aspirin use was associated with a higher risk of acute myocardial infarction, ischemic stroke, and cardiovascular mortality. Second, though statins may have positive effects on bone formation,^34^ uncontrolled confounding is the best explanation for the reduced risk of fractures that emerges after 2 years of continued statin use.^25^ Our conclusion is in line with a recent meta-analysis, in which statin use was associated with a lower risk of fractures in observational studies, but not in RCTs.^35^ Third, statin use seemed to have a very strong protective effect on mortality, and especially liver-related mortality, after just 6–12 months of use. Though it is in agreement with meta-analyses of observational studies 36,37 and the few small RCTs on the subject,^38^,^39^ such a strong effect is implausible. Indeed, despite our design, this may be explained by statin users being “healthier” than nonusers in ways that we could not capture with this study. In contrast, though HCC and other cancers do not share the same risk factors, the null association between statin use and non-HCC risk suggests that some common risk factors for cancer (e.g., smoking) were well-balanced. Furthermore, immortal time bias was eliminated by specifying statin initiation before the start of follow-up,^40^ and protopathic bias was minimized by applying a lag-time in censoring by protocol deviation.^21^ Last, plenty of possible biologic mechanisms for the protective effect of statins backed by experimental results have been proposed.^41^ Taken together, it remains possible that statins have beneficial effects on HCC risk and on death without HCC, however, we believe that we overestimated the potential effect size.

The same issues of unmeasured and unmeasurable confounding apply to the aspirin findings, and the observed null effect of aspirin could be due to uncontrollable confounding in the negative direction. This possibility is supported by the fact that patients in the aspirin cohort had a slightly elevated risk of non-HCC cancer and fractures. However, we deem it more likely that aspirin has no effect on the risk of HCC or on the risk of death without HCC, and we suggest that previously reported beneficial effects of aspirin were due to uncontrolled bias.

Existing studies of the effects of statins and/or aspirin on the risk of HCC can be grouped into 3 categories: (1) case-control studies of the odds of having used statins or aspirin,^4^,^5^ (2) cohort studies of the effect of initiating treatment in which the exposure is only defined at baseline,^6^,^7^ and (3) cohort studies in which both the exposure and the confounders are time-varying.^8^,^9^ There are a few possible explanations to the discrepant effects of aspirin in our study and in previous studies. First, studies showing a null effect of aspirin are less likely to be published (publication bias), and second, the published studies might simply suffer from the same biases: Most notably, (1) the case-control studies are prone to temporal and selection biases, (2) the baseline cohort studies are prone to confounding by indication and immortal time bias, and (3) the time-varying cohort studies are prone to healthy user effects, protopathic bias, and time-varying confounding. Furthermore, most studies have not utilized a new user design, and prior studies have not specified the minimally sufficient adjustment set of confounders.^19^ Two prior studies have investigated the effects of both statins and aspirin, and in both cases, the effect of statins was more protective.^5^,^6^ For example, in one study, the adjusted odds ratio for HCC risk was 0.34 (95% CI: 0.32–0.37) for statins and 0.92 (95% CI: 0.85–0.99) for aspirin.^5^ Though they did not find a null effect of aspirin, this observed difference between the statin effect and the aspirin effect is in line with our findings. Of note, our study was conducted in patients with ALD cirrhosis, and our findings may not generalize to patients with other liver diseases.

The clinical takeaway from our study is that aspirin is not indicated for the chemoprevention of HCC and that it is impossible to determine the true chemopreventive effect of statins without an RCT. Despite the use of sophisticated statistical/epidemiologic methods to eliminate bias and confounding, we cannot rule out residual bias. Clinicians must also consider the safety of statins. An RCT from 2019 found that treatment with 40 mg/day compared with 20 mg/day caused more adverse events in patients with decompensated cirrhosis.^42^ Statins may potentially have a beneficial effect, but with the current knowledge, we recommend that clinicians do not use statins to prevent HCC or to reduce HCC-free mortality. However, beyond any liver-specific aims, clinicians should continue to prescribe these drugs to reduce the cardiovascular risk in patients with ALD cirrhosis.

In conclusion, compared with 5 years of nonuse, 5 years of continued statin use was associated with a reduced risk of HCC and of death without HCC in patients with ALD cirrhosis. However, this apparent effect of statins is fully or partly explained by uncontrollable confounding, and nothing short of an RCT can settle the issue. Aspirin likely has no effect on the risk of HCC or death without HCC in this population. Based on our findings, neither statins nor aspirin are indicated to prevent HCC or reduce mortality among patients with ALD cirrhosis.

## Supplementary Material
