# Supplementary material for: Effects of statins and aspirin on HCC risk in alcohol-related cirrhosis: nationwide emulated trials
Source: Hepatol Commun. 2023 Jan 3;7(1):e0013. doi: 10.1097/HC9.0000000000000013 (PMC9827970; doi:10.1097/HC9.0000000000000013)
Supplement: Supplementary file 1 [file hc9-7-e0013-s001.docx]

Supplementary material

Effects of statins and aspirin on hepatocellular carcinoma risk in alcohol-related cirrhosis: nationwide emulated trials

Frederik Kraglund, Diana Hedevang Christensen, Andreas Halgreen Eiset, Gerda Elisabeth Villadsen, Joe West, Peter Jepsen

**Table of contents**

[Supplementary methods 2](#_Toc116285609)

[Setting up the data 2](#_Toc116285610)

[Inverse probability weighting 2](#_Toc116285611)

[Risk estimates 2](#_Toc116285612)

[Supplementary Figure S1 4](#_Toc116285613)

[Supplementary Figure S2 5](#_Toc116285614)

[Supplementary Figure S3 6](#_Toc116285615)

[Supplementary Figure S4 7](#_Toc116285616)

[Supplementary Figure S5 8](#_Toc116285617)

[Supplementary Figure S6 9](#_Toc116285618)

[Supplementary Table S1 10](#_Toc116285619)

[Supplementary Table S2 12](#_Toc116285620)

[References 14](#_Toc116285621)

# Supplementary methods

### Setting up the data

The method used in this study to construct the marginal structural models using inverse probability weighting is the same used by Dickerman et al (1). Marginal structural models require discrete time intervals with a suitable number of outcomes occurring during each interval. Half-year intervals were chosen because enough outcomes occurred during each interval to ensure statistical stability, while the outcome was still rare enough for logistic regression to approximate Cox regression (2). Thus, data was set up with one observation for each time interval contributed by each patient in each trial. Hepatocellular carcinoma (HCC) was the outcome of interest, and death without HCC was a competing event. Patients were followed until HCC, death without HCC, protocol deviation, end of trial (after 5 years), or administrative censoring on 31 December 2018.

### Inverse probability weighting

The probabilities used to calculate the stabilized inverse probability of treatment weights (SIPTW) and the stabilized inverse probability of censoring weights (SIPCW) were computed using logistic regression. The SIPTWs for at given trial arm were calculated as the probability of being included in the given trial-arm divided by the probability of being included in the given trial-arm given baseline confounders (3). Standardized mean differences (SMDs) were used to assess whether the baseline confounders were well-balanced after applying the SIPTWs (4). The time-varying SIPCWs were calculated in two steps: First, the SIPCW was calculated for each time interval in a given trial-arm as the probability of deviating from protocol given baseline confounders divided by the probability of deviating from protocol given baseline *and* time-varying confounders. Second, the time-varying SIPCW for a given **time** interval was calculated as the product of the SIPCWs for that and all preceding time intervals (5). The SIPTWs and the time-varying SIPCWs were multiplied for each time interval to obtain the final weights. These weights, when applied, creates a pseudo-population in which the probability of being included in a given trial-arm and remaining uncensored is independent of the specified confounders.

### Risk estimates

The method used in this study to obtain cumulative risk estimates from marginal structural models is the same used by Jepsen et al (6). The cumulative risks and relative risk of HCC (and, separately, death without HCC) was estimated for the exposed and the unexposed trial arms representing the two treatment strategies: 1) use statins (or, separately, aspirin) continuously for five years, and 2) do not use statins (aspirin) for five years. These risk estimates were computed in a stepwise approach. First, the probability of HCC (and, separately, of death without HCC) was computed for each time interval using logistic regression weighted by baseline SIPTW and time-varying SIPCW and also adjusted for baseline confounders (doubly robust estimation(7)). Second, to account for competing risks, the probabilities of HCC and of death without HCC during a given time interval were multiplied by the probability of being alive and HCC-free at the beginning of that time interval (8). Third and last, the *cumulative* risk estimates for a given time interval were calculated by adding the risk estimate of current time interval to those of each preceding time interval. The relative risk estimates were then derived by simple division of the cumulative risk in the exposed arm by the cumulative risk in the unexposed arm. Because the half-yearly event-rate was low, and because competing risks were accounted for, the obtained relative risk estimates approximate subdistribution hazard ratios (2). Percentile-based 95% confidence intervals (95% CIs) were computed using nonparametric cluster bootstrapping with 500 replications, where each cluster represented an individual patient.

Supplementary Figure S1**.** The presumed causal relationships between statin treatment, HCC, death without HCC, and the confounding covariates. Green labels represent unmeasured covariates, grey labels represent measured covariates, and black labels represent outcomes.


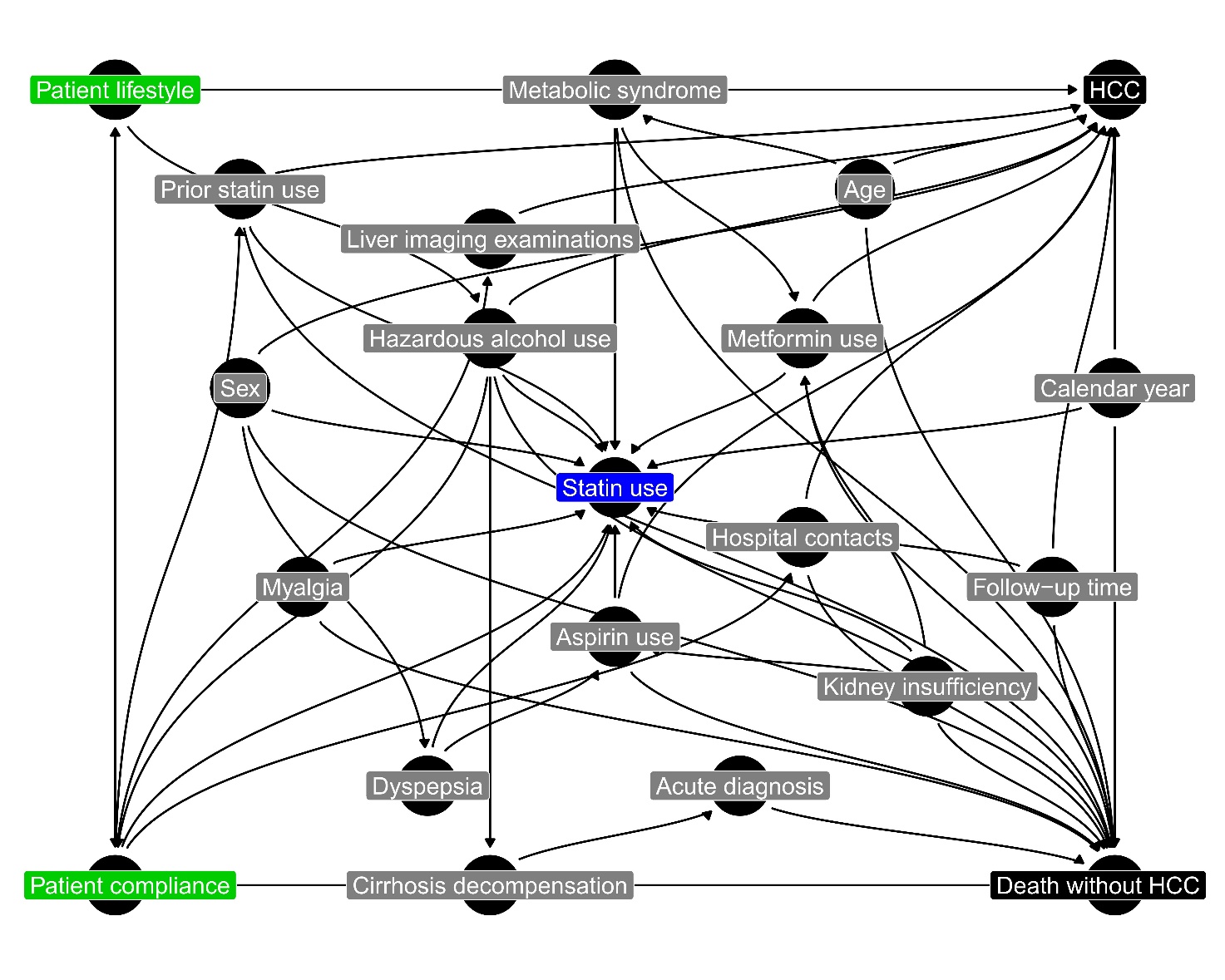


Supplementary Figure S2**.** The presumed causal relationships between aspirin treatment, HCC, death without HCC, and the confounding covariates. Green labels represent unmeasured covariates, grey labels represent measured covariates, and black labels represent outcomes.


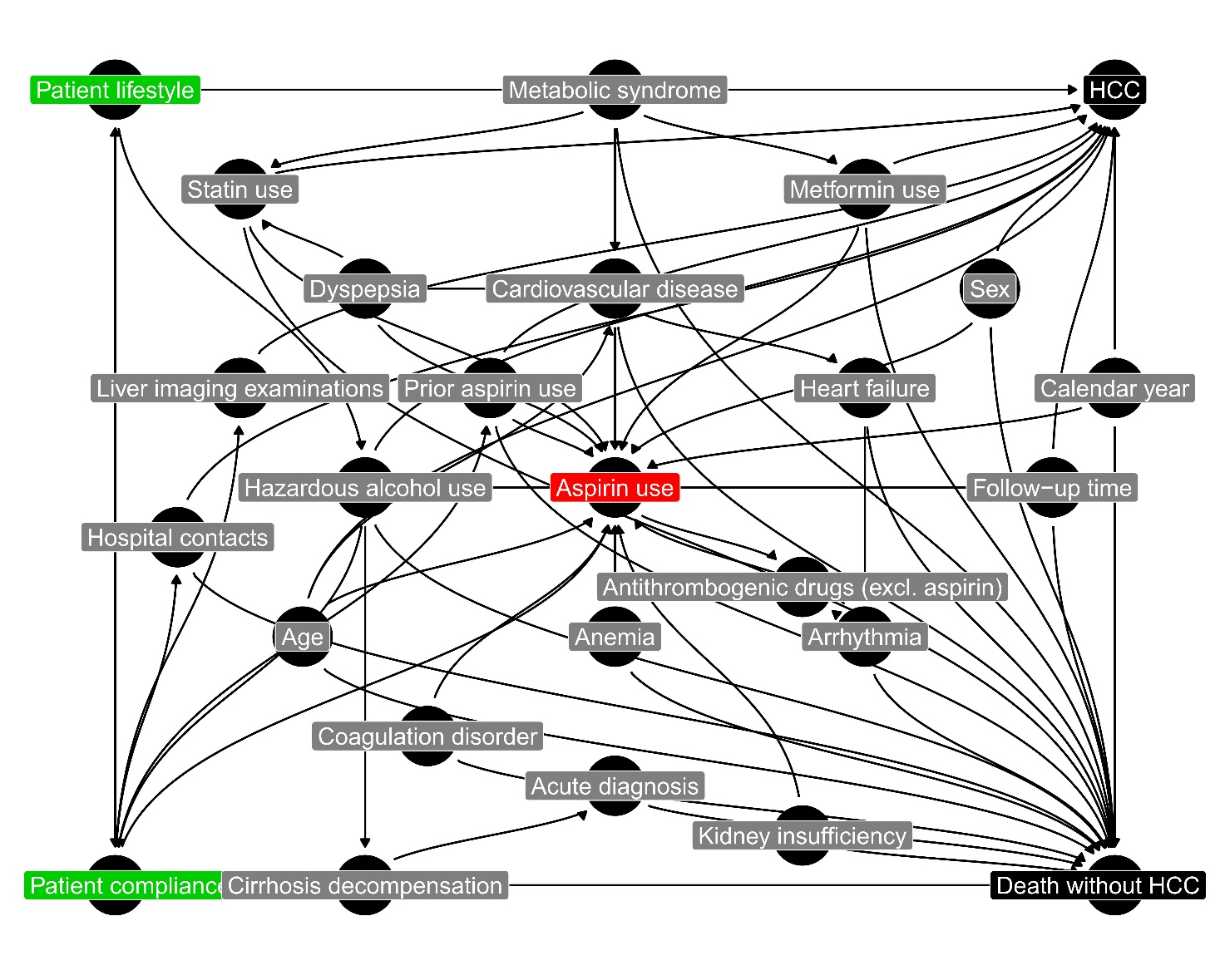


Supplementary Figure S3**.** Illustrations of the study design. Examples: patient 2 in trial 1, patient 4 in trials 1 and 2, and patient 5 in trial 2 exemplify censoring due to protocol deviation within the unexposed trial arm; and patient 4 in trial 3 exemplifies censoring due to protocol deviation in the exposed arm.


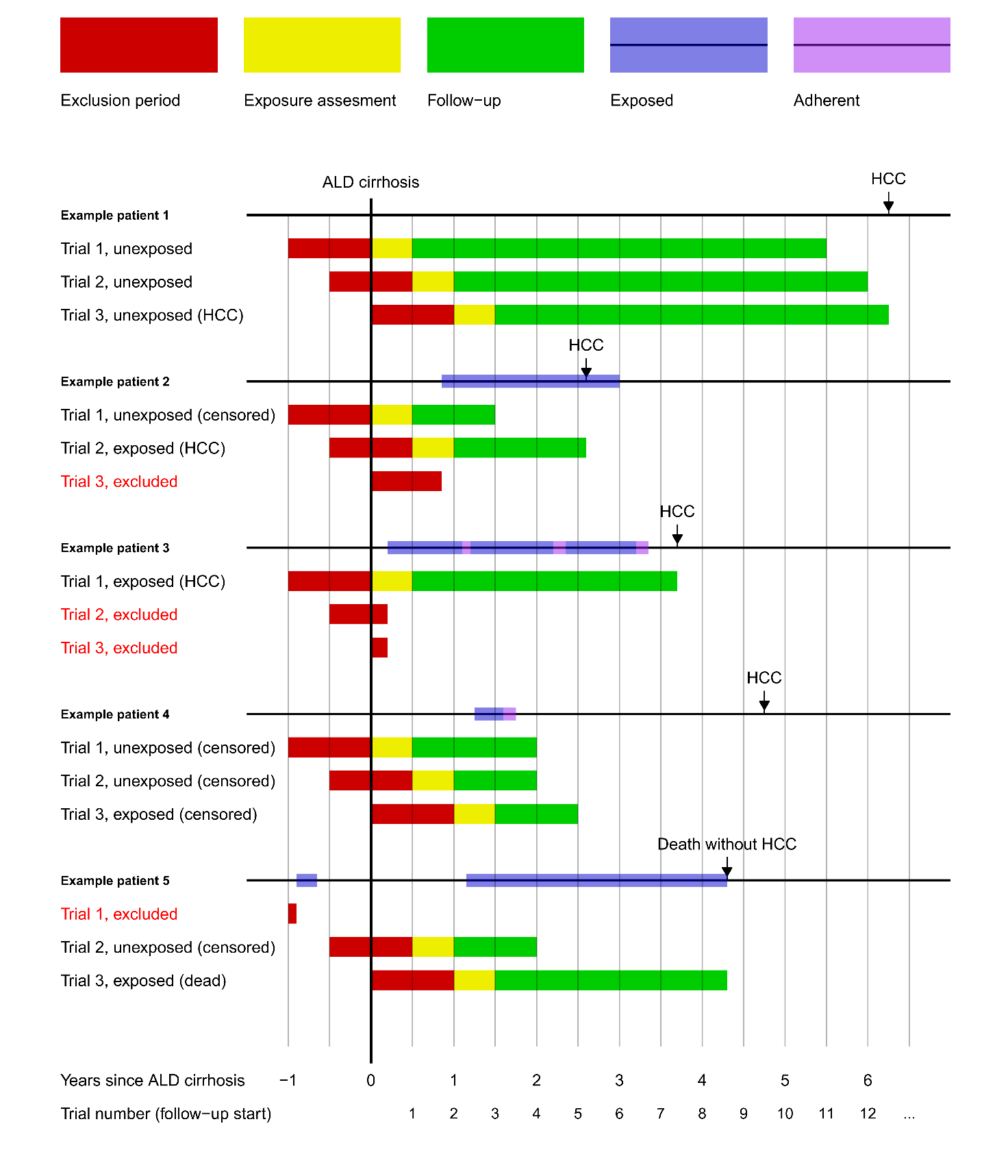


Supplementary Figure S4**.** The effect of statins on control outcomes (non-HCC cancer, fractures commonly caused by low-energy trauma, lung cancer, and acute myocardial infarction or ischemic stroke) shown along with the primary outcomes as the cumulative incidence in the exposed and unexposed arms. Abbreviations; AMI, acute myocardial infarction.


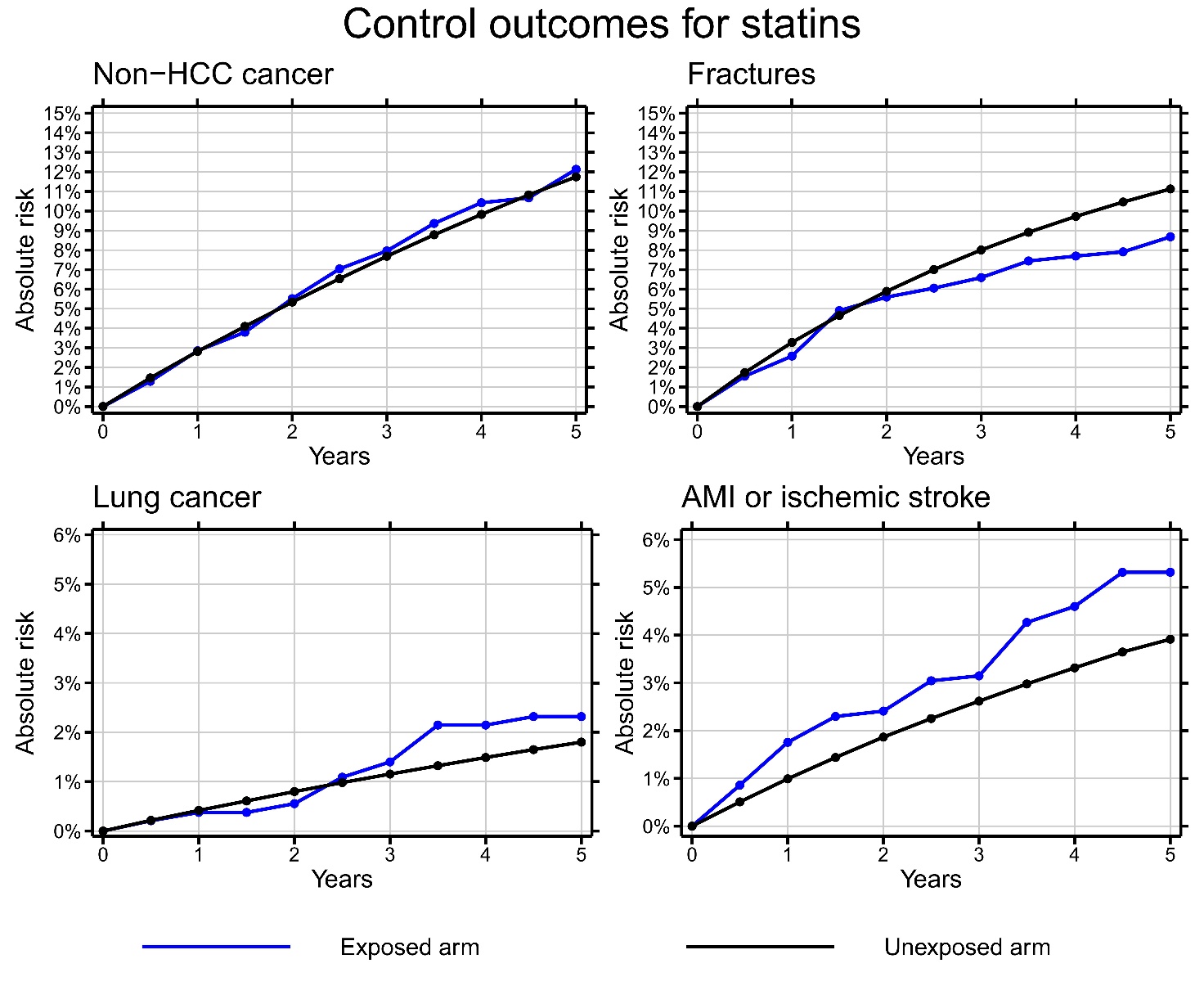


Supplementary Figure S5**.** The effect of aspirin on control outcomes (non-HCC cancer, fractures commonly caused by low-energy trauma, lung cancer, and acute myocardial infarction or ischemic stroke) shown along with the primary outcomes as the cumulative incidence in the exposed and unexposed arms. Abbreviation: AMI, acute myocardial infarction.


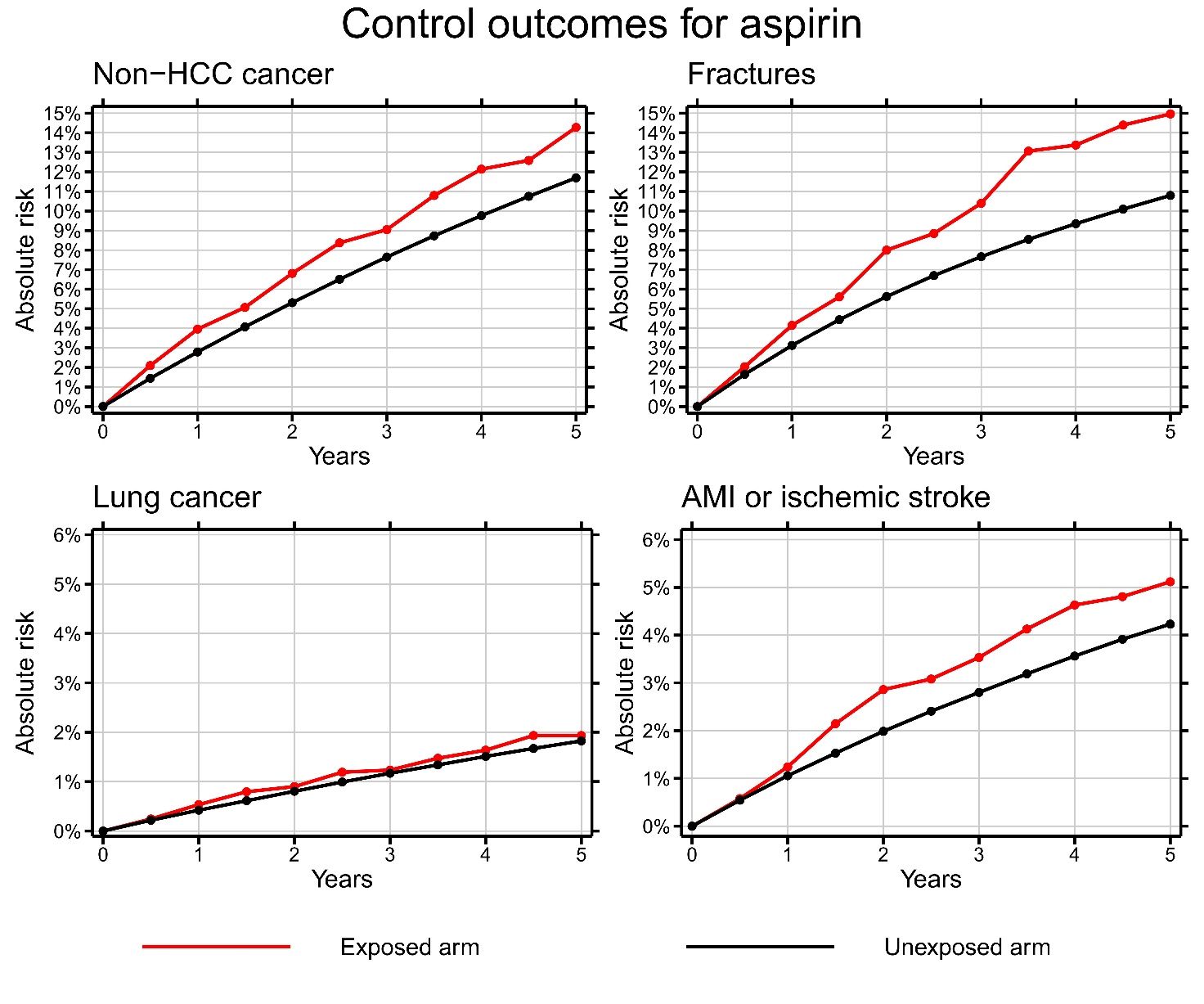


Supplementary Figure S6**.** The effect of statins (left) and aspirin (right) on the cumulative incidence of HCC (top) and of death without HCC (bottom) after excluding the first year of follow-up.


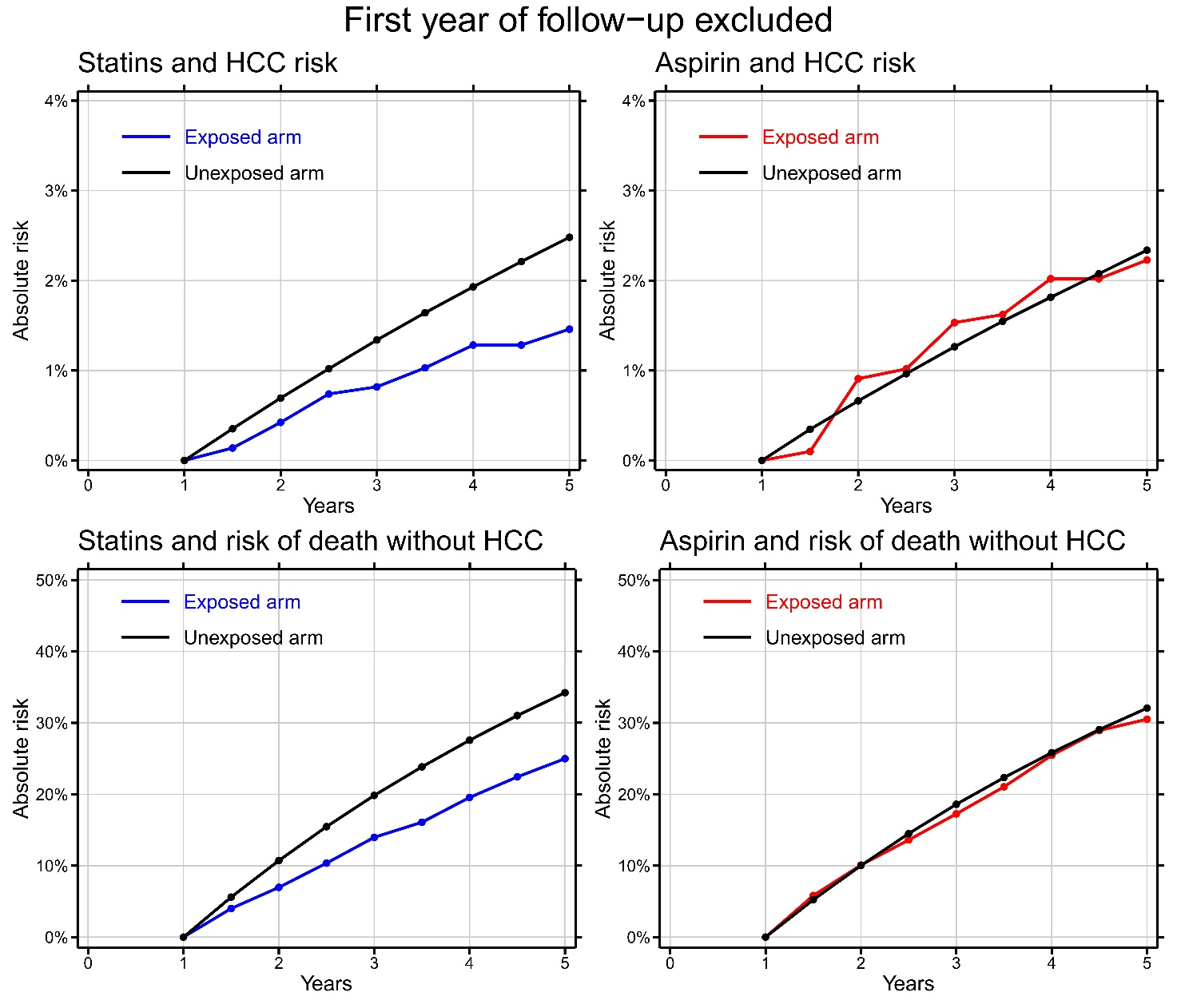


Supplementary Table S1**.** Covariates included in the model to remove confounding between the effect of statins on HCC and/or death without HCC.

| **Variable** | **Measure** | **Definition** | **Subdefinitions** | **Codes** |
| --- | --- | --- | --- | --- |
| **Basic characteristics** | | | | |
| Age | Four categories | Age at time of inclusion |  |  |
| Sex | Binary | Sex |  |  |
| Calendar year | Three categories | Calendar year at time of inclusion |  |  |
| Follow-up time | Natural cubic spline with five knots at 5th, 27.5th, 50th, 72.5th and 95th percentiles | Time since trial inclusion |  |  |
| Trial | Continuous | Trial number (time since first diagnosis of cirrhosis due to alcohol-related liver disease) |  |  |
| Cumulative prior use of statins | Five categories | Cumulative use of statins prior to one and a half years before inclusion | Statins | ATC: C10AAx, C10BAx, C10BXx |
| ****Administrative characteristics**** | | | | |
| Hospital contacts | Continuous | Number of hospital contacts in the year leading up to inclusion |  |  |
| Liver imaging examination | Binary | Whether the patient had a liver imaging examination in the year leading up to inclusion | Liver imaging examinations | DCE: UXCD00, UXCD10 UXCD40, UXMD10 UXMD40, UXUD05, UXUD10, UXUD11, UXUD50, UXUD70 |
| Acute diagnosis | Binary | Whether the first ALD cirrhosis diagnosis was given during acute admission or not |  |  |
| ****Relative statin contraindications**** | | | | |
| Decompensation | Binary | Prior cirrhosis decompensation |  |  |
|  |  |  | Ascites | ICD-10: R18x |
|  |  |  | Spontaneous bacterial peritonitis | ICD-10: K658I |
|  |  |  | Oesophageal variceal bleeding | ICD-10: I850 |
|  |  |  | Gastric variceal bleeding | ICD-10: I864A |
|  |  |  | Hepatorenal syndrome | ICD-10: K767 |
|  |  |  | Treatment of oesophageal varices | NCSP: KJCA20, KJCA22, KJCA32 |
|  |  |  | Ascites drainage | NCSP: KTJA10x |
| Kidney insufficiency | Binary | Prior kidney insufficiency | Kidney insufficiency | ICD-10: N17x-N19x |
| **Metabolic syndrome-related diseases** | | | | |
| Hyperlipidaemia | Binary | Prior hyperlipidaemia | Hyperlipidaemia | ICD-10: E780x-E785x, E789 |
| Obesity | Binary | Prior obesity |  |  |
|  |  |  | Obesity diagnosis | ICD-10: E65x, E66x, E68x, E888C |
|  |  |  | Anti-obesity drugs | ATC: A08x, A10BJ02 (Saxenda^®^) |
| Hypertension | Binary | Prior hypertension |  |  |
|  |  |  | Hypertension diagnosis | ICD-10: I10x-I15x |
|  |  |  | Antihypertensive drugs | ATC: C02x, C07x-C09x |
| Diabetes | Binary | Prior diabetes |  |  |
|  |  |  | Diabetes diagnoses | ICD-10: E10x-E14x |
|  |  |  | Antidiabetic drugs | ATC: A10x |
|  |  |  | Diabetes complication diagnoses | ICD-10: G632, H360x, N083 |
| **Heart diseases** | | | | |
| Cardiovascular disease | Binary | Prior cardiovascular disease |  |  |
|  |  |  | Atherosclerosis | ICD-10: I251, K551A, I672, I70x |
|  |  |  | Thromboembolism | ICD-10: H341A, I272x, I513x, I63x (excl. I632 and I635), I74x, K550H, K550C, N280A, N280D, T817Bx, T823D, T828A, Z867B |
|  |  |  | Ischemic heart disease | ICD-10: I20x-I25x |
|  |  |  | Thrombosis treatments | NCSP: KFNx, KFLFx, KPBE, KPBF, KPBH, KPBN, KPBP, KPBQ, KPBW, KPEE, KPEF, KPEH, KPEN, KPEP, KPEQ, KPEU74, KPEU82, KPEU83, KPEU84, KPEW, KPFE, KPFH, KPFN, KPFP, KPFQ, KPFU74, KPFU82, KPFU83, KPFU84, KPFW, KPWG, KAAL10, KAAL11, KPAE, KPAF, KPAH, KPAN, KPAP, KPAQ, KPAW99, KPAU74,KPCE, KPCF, KPCH, KPCN, KPCP, KPCQ, KPCW99, KPCW20, KPCU74, KPCU82, KPCU83, KPCU84, KPDE, KPDF, KPDH, KPDN, KPDP, KPDQ, KPDU74, KPDU82, KPDU83, KPDU84, KPDW99, KPDW20 |
| **Common statin side effects** | | | | |
| Dyspepsia | Binary | Prior dyspepsia |  |  |
|  |  |  | Upper gastrointestinal diseases (incl. peptic ulcer and functional dyspepsia) | ICD-10: K20x-K31x |
|  |  |  | Proton pump inhibitor use | ATC: A02BCx |
|  |  |  | Antacid use | ATC: A02Ax |
| Myalgia | Binary | Prior myalgia |  |  |
|  |  |  | Myopathy | ICD-10: M60x-M63x |
|  |  |  | Myalgia | ICD-10: M790, M791, M797 |
| **Other potential confounders** | | | | |
| Metformin | Binary | Metformin use | Metformin | ATC: A10BAx |
| Aspirin | Binary | Aspirin use | Aspirin | ATC: B01AC06 |
| Hazardous alcohol use | Binary | Hazardous alcohol use | Hazardous alcohol use relapse | ICD-10: F10.0x-F10.1x, F10.2, F10.24-F10.26, F10.3x-F10.5x |

Abbreviations: ALD cirrhosis, cirrhosis due to alcohol-related liver disease; DCE, Danish Classification of Examinations; ICD-10, International Classification of Diseases - 10th revision; SNOMED, Systemized Nomenclature of Medicine; NCSP, NOMESCO Classification of Surgical Procedures; ATC, Anatomical Therapeutic Chemical

Supplementary Table S2**.** Covariates included in the model to remove confounding between the effect of aspirin on HCC and/or death without HCC.

| **Variable** | **Measure** | **Definition** | **Subdefinitions** | **Codes** |
| --- | --- | --- | --- | --- |
| **Basic characteristics** | | | | |
| Age | Four categories | Age at time of inclusion |  |  |
| Sex | Binary | Sex |  |  |
| Calendar year | Three categories | Calendar year at time of inclusion |  |  |
| Follow-up time | Natural cubic spline with five knots at 5th, 27.5th, 50th, 72.5th and 95th percentiles | Time since trial inclusion |  |  |
| Trial | Continuous | Trial number (time since first diagnosis of cirrhosis due to alcohol-related liver disease) |  |  |
| Cumulative prior use of aspirin | Five categories | Cumulative use of aspirin prior to one and a half years before trial inclusion | Aspirin | ATC: B01AC06 |
| **Administrative characteristics** | | | | |
| Hospital contacts | Continuous | Number of hospital contacts in the year prior to the first ALD cirrhosis diagnosis |  |  |
| Liver imaging examinations | Binary | Whether the patient had a liver imaging examination in the year leading up to inclusion | Liver imaging examinations | DCE: UXCD00, UXCD10 UXCD40, UXMD10 UXMD40, UXUD05, UXUD10, UXUD11, UXUD50, UXUD70 |
| Acute diagnosis | Binary | Whether the first ALD cirrhosis diagnosis was given during acute admission or not |  |  |
| **Relative aspirin contraindications** | | | | |
| Decompensation | Binary | Prior cirrhosis decompensation |  |  |
|  |  |  | Ascites | ICD-10: R18x |
|  |  |  | Spontaneous bacterial peritonitis | ICD-10: K658I |
|  |  |  | Esophageal variceal bleeding | ICD-10: DI850 |
|  |  |  | Gastric variceal bleeding | ICD-10: DI864A |
|  |  |  | Hepatorenal syndrome | ICD-10: K767 |
|  |  |  | Treatment of esophageal varices | NCSP: KJCA20, KJCA22, KJCA32 |
|  |  |  | Ascites drainage | NCSP: KTJA10x |
| Kidney insufficiency | Binary | Prior kidney insufficiency | Kidney insufficiency | ICD-10: N17x-N19x |
| **Absolute aspirin contraindications** | | | | |
| Coagulation deficiency | Binary | Prior coagulation deficiency | Coagulation deficiency | ICD-10: D65x-D69x |
| **Metabolic syndrome-related diseases** | | | | |
| Hypertension | Binary | Prior hypertension |  |  |
|  |  |  | Hypertension diagnosis | ICD-10: I10x-I15x |
|  |  |  | Antihypertensive drugs | ATC: C02x, C07x-C09x |
| Diabetes | Binary | Prior diabetes |  |  |
|  |  |  | Diabetes diagnoses | ICD-10: E10x-E14x |
|  |  |  | Antidiabetic drugs | ATC: A10x |
|  |  |  | Diabetes complication diagnoses | ICD-10: G632, H360x, N083 |
| **Heart diseases** | | | | |
| Cardiovascular disease | Binary | Prior cardiovascular disease |  |  |
|  |  |  | Atherosclerosis | ICD-10: I251, K551A, I672, I70x |
|  |  |  | Thromboembolism | ICD-10: H341A, I272x, I513x, I63x (excl. I632 and I635), I74x, K550H, K550C, N280A, N280D, T817Bx, T823D, T828A, Z867B |
|  |  |  | Ischemic heart disease | ICD-10: I20x-I25x |
|  |  |  | Thrombosis treatments | NCSP: KFNx, KFLFx, KPBE, KPBF, KPBH, KPBN, KPBP, KPBQ, KPBW, KPEE, KPEF, KPEH, KPEN, KPEP, KPEQ, KPEU74, KPEU82, KPEU83, KPEU84, KPEW, KPFE, KPFH, KPFN, KPFP, KPFQ, KPFU74, KPFU82, KPFU83, KPFU84, KPFW, KPWG, KAAL10, KAAL11, KPAE, KPAF, KPAH, KPAN, KPAP, KPAQ, KPAW99, KPAU74,KPCE, KPCF, KPCH, KPCN, KPCP, KPCQ, KPCW99, KPCW20, KPCU74, KPCU82, KPCU83, KPCU84, KPDE, KPDF, KPDH, KPDN, KPDP, KPDQ, KPDU74, KPDU82, KPDU83, KPDU84, KPDW99, KPDW20 |
| Heart failure | Binary | Prior heart failure | Heart failure | ICD-10: I50x |
| Heart arrhythmia | Binary | Prior heart arrhythmia | Heart arrhythmia | ICD-10: I44x-I49x |
| **Aspirin side effects** | | | | |
| Dyspepsia | Binary | Prior dyspepsia |  |  |
|  |  |  | Upper gastrointestinal diseases (incl. peptic ulcer and functional dyspepsia) | ICD-10: K20x-K31x |
|  |  |  | Proton pump inhibitor use | ATC: A02BCx |
|  |  |  | Antacid use | ATC: A02Ax |
| Anemia | Binary | Prior anemia | Anemia | ICD-10: D50x-D53x, D55x-D64x |
| **Other potential confounders** | | | | |
| Metformin | Binary | Metformin use | Metformin | ATC: A10BAx |
| Statins | Binary | Statin use | Statins | ATC: C10AAx, C10BAx, C10BXx |
| Antithrombogenic drugs (except aspirin) | Binary | Use of oral anticoagulants and antithrombotic drugs (except aspirin) | Antithrombogenic drugs | ATC: B01AAx, B01ACx (excl. B01AC06), B01AEx, B01AFx, B01AX05 |
| Hazardous alcohol use | Binary | Hazardous alcohol use | Hazardous alcohol use relapse | ICD-10: F10.0x-F10.1x, F10.2, F10.24-F10.26, F10.3x-F10.5x |

Abbreviations: ALD cirrhosis, cirrhosis due to alcohol-related liver disease; DCE, Danish Classification of Examinations; ICD-10, International Classification of Diseases - 10th revision; SNOMED, Systemized Nomenclature of Medicine; NCSP, NOMESCO Classification of Surgical Procedures; ATC, Anatomical Therapeutic Chemical

# References

1. Dickerman BA, García-Albéniz X, Logan RW, Denaxas S, Hernán MA. Avoidable flaws in observational analyses: an application to statins and cancer. Nat Med 2019;25:1601-1606.

2. Thompson WA, Jr. On the treatment of grouped observations in life studies. Biometrics 1977;33:463-470.

3. Robins JM, Hernán MÁ, Brumback B. Marginal Structural Models and Causal Inference in Epidemiology. Epidemiology 2000;11.

4. Austin PC, Stuart EA. Moving towards best practice when using inverse probability of treatment weighting (IPTW) using the propensity score to estimate causal treatment effects in observational studies. Stat Med 2015;34:3661-3679.

5. Fewell Z, Hernán MA, Wolfe F, Tilling K, Choi H, Sterne JAC. Controlling for Time-dependent Confounding using Marginal Structural Models. The Stata Journal 2004;4:402-420.

6. Dam G, Vilstrup H, Andersen PK, Bossen L, Watson H, Jepsen P. Effect of proton pump inhibitors on the risk and prognosis of infections in patients with cirrhosis and ascites. Liver Int 2019;39:514-521.

7. Funk MJ, Westreich D, Wiesen C, Stürmer T, Brookhart MA, Davidian M. Doubly robust estimation of causal effects. Am J Epidemiol 2011;173:761-767.

8. Fine JP, Gray RJ. A Proportional Hazards Model for the Subdistribution of a Competing Risk. Journal of the American Statistical Association 1999;94:496-509.
